# Microsaccades enable efficient synchrony-based coding in the retina: a simulation study

**DOI:** 10.1038/srep24086

**Published:** 2016-04-11

**Authors:** Timothée Masquelier, Geoffrey Portelli, Pierre Kornprobst

**Affiliations:** 1 INSERM, U968, Paris, F-75012, France; 2 Sorbonne Universités, UPMC Univ Paris 06, UMR_S 968, Institut de la Vision, Paris, F-75012, France; 3 CNRS, UMR_7210, Paris, F-75012, France; 4 Biovision Team, Inria Sophia Antipolis Méditerranée, 06902, France

## Abstract

It is now reasonably well established that microsaccades (MS) enhance visual perception, although the underlying neuronal mechanisms are unclear. Here, using numerical simulations, we show that MSs enable efficient synchrony-based coding among the primate retinal ganglion cells (RGC). First, using a jerking contrast edge as stimulus, we demonstrate a qualitative change in the RGC responses: synchronous firing, with a precision in the 10 ms range, only occurs at high speed and high contrast. MSs appear to be sufficiently fast to be able reach the synchronous regime. Conversely, the other kinds of fixational eye movements known as tremor and drift both hardly synchronize RGCs because of a too weak amplitude and a too slow speed respectively. Then, under natural image stimulation, we find that each MS causes certain RGCs to fire synchronously, namely those whose receptive fields contain contrast edges after the MS. The emitted synchronous spike volley thus rapidly transmits the most salient edges of the stimulus, which often constitute the most crucial information. We demonstrate that the readout could be done rapidly by simple coincidence-detector neurons without knowledge of the MS landing time, and that the required connectivity could emerge spontaneously with spike timing-dependent plasticity.

Our eyes are never at rest. Even when trying to fixate, we make involuntary and unconscious eye movements. These movements, known as fixational eye movements (FEM), have three main components: tremor, drift, and microsaccades (MS)^1^,^2^,^3^. Tremor is an aperiodic, wave-like motion of the eyes with a frequency of ~90 Hz. It is the smallest of all eye movements, with an amplitude of only about the diameter of a cone in the fovea. Drift is a slow and random-walk-like movement that occurs, together with the tremor, between the MSs. MSs are rapid ballistic jumps of amplitude up to 1°, which occur at irregular intervals, only once or twice per second. They carry the retinal image over dozens of photoreceptor width or more, in only 20–30 ms^1^,^2^.

Some experiments suggest that MSs enhance visual perception by counteracting its fading^1^,^2^,^3^, although the drift alone could be sufficient do so^4^,^5^. Furthermore, even if MSs are not voluntary, we do more of them when paying attention^6^, in particular to fine spatial details^7^,^8^, or when looking at informative regions^9^, suggesting that MSs are involved in active sensing loops. This calls for an explanation at the neuronal level. The prevalent view is that MSs counteract the retinal ganglion cell (RGC) adaptation to unchanging stimuli, generating strong transient responses. Here we suggest that in addition to the increase in spiking activity, MSs may cause some RGCs to fire synchronously, and that the subset of synchronous RGCs may be a signature of the underlying visual stimulus’ edges (i.e. its high spatial frequency content).

To the best of our knowledge, Greschner and colleagues are the only ones who have studied the relationship between FEM and RGC synchrony^10^. They found that in the turtle retina, a 5 Hz periodic movement with an amplitude of about one photoreceptor width caused the RGCs with receptive fields (RF) located along contrast edges to synchronize^10^. These movements, however, are quite different from MSs in primates, which, as previously stated, occur more rarely (once or twice per second), at irregular intervals, and rapidly carry the retinal image over much longer distances. In addition, the study by Greschner and colleagues did not address the problem of synchrony-based encoding of natural images.

Here, using numerical simulations, we show that the MSs, being in the proper range of speed and amplitude, are *sufficient* to synchronize a small subset of RGCs, namely those that are strongly activated by the image content corresponding to the MS landing location. Conversely, tremor and drift both hardly synchronize RGCs because of a too weak amplitude and a too slow speed respectively. We thus suggest a new role for MSs: to allow synchrony-based coding in the retina. This differs from earlier proposals such as time-to-first-spike coding with respect to MS landing times, or rank-order coding^11^, which are possible in theory as the brain “knows” when it generates MSs^3^.

In this paper, by “synchrony” we mean the co-occurrence of spikes within a time window sufficiently narrow to contain a maximum of one spike per cell, e.g. ~10 ms. This contrasts with “rate co-modulation”, which is the cross-cell correlation of spike counts within broader time windows^12^. In addition, the focus is on stimulus-driven synchrony^13^,^14^: e.g. two distant RGCs with non-overlapping RFs yet displaying responses that are modulated by the same stimuli in a similar and simultaneous fashion.

## Results

We used the Virtual Retina software^15^, which allows large-scale simulations of biologically-plausible retinas, with customizable parameters. Virtual Retina has been shown to reproduce a wide range of experimental data from salamander, cat and primate retinas^15^, and has been used in several theoretical studies^16^,^17^,^18^,^19^. It has recently been shown to predict spikes in a mouse retina more accurately than linear-nonlinear (LN) models^20^. The underlying model includes a non-separable spatio-temporal linear model of filtering in the Outer Plexiform Layer, a shunting feedback at the level of bipolar cells, and a spike generation process using noisy leaky integrate-and-fire neurons to model RGCs. All parameters for the different stages of the model are customizable so that the visual field can be paved with different RGC types.

Here, we modeled the foveal region of a primate retina and only included the midget cells, which represent about 95% of the foveal RGCs^21^. These cells presumably mediate high-acuity vision for static stimuli^22^. They feed the parvocellular layers of the lateral geniculate nucleus (LGN), which constitute the main input for the ventral stream of the visual cortex^23^, involved in form representation and object recognition^24^. Midget cells have classic center-surround RFs, and are of two main types: ON-cells which are excited by bright dots surrounded by dark regions, whereas OFF-cells are excited by dark dots surrounded by bright regions. Midget cells have strong transient (phasic) responses and weaker sustained (tonic) ones. All the model parameters are given in Table 1.

### Synchronous firing needs fast jerky motions and high contrast

**Figure 1:**
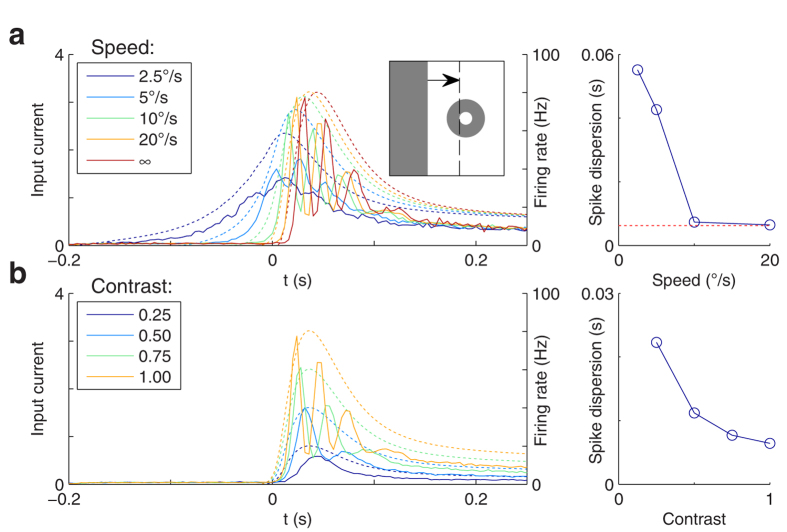
Effect of speed and contrast of an arriving contrast edge on spike time precision. (**a**-left) The inset shows the stimulation scheme: a dark edge enters the RF of an ON-center RGC and stops at the border with the center area. The main plot shows, for the different speeds of the moving edge, the RGC’s input current (dotted lines; units: threshold current), and the resulting PSTH (solid lines), aligned so that the edge stops at t = 0 s. Infinite speed is the limit case, meaning that the edge appears directly in its final position. Notice that during the transient response, spiking is synchronous for speeds of 10°/s and above, and asynchronous for slower speeds. Conversely, the sustained response, say for t > 0.15 s, is always asynchronous. (**a**-right) Spike time dispersion as a function of speed. These dispersions are estimated by fitting the PSTHs with Gaussian mixture models (see Supplementary Figure S1). The horizontal dotted line shows the infinite-speed asymptote, which is almost reached for speeds of 10°/s and above. (**b**-left) The RGC’s response for different contrast values (and with a speed of 20°/s) (**b**-right) Spike time dispersion as a function of contrast. Only strong contrasts cause precise spike times.

We first investigated the conditions under which jerk-like motions may generate synchronous firing. Let us consider the subset of RGCs that are strongly activated after one jerk, presumably due to the presence of contrast edges in their RFs. Likely, most of these cells were previously exposed to homogeneous regions before the jerk, as these are much more common than edges in ecological visual environments. With this observation in mind, we started with a simple scenario: we examined the response of a single ON-cell when, after being exposed to a bright homogeneous surface, a dark edge penetrates its OFF-surround area and stops at the border with its ON-center area, thus strongly stimulating the cell (Fig. 1a left, inset). Responses of that cell are shown in Fig. 1 for different values of penetrating speed and contrast. When varying the speed, the evoked input current peak grows monotonously with the speed (Fig. 1a left, dotted lines). Yet we observe a qualitative change in the firing behavior. At low speeds (<10°/s), the peristimulus time histograms (PSTH, solid lines) simply reflect the input fluctuations. Conversely, at higher speeds spike times become reproducible across trials, leading to sharp peaks in the PSTH. We define the spike time dispersion as the width of the first PSTH peak, estimated by fitting the PSTH with Gaussian mixture models (see Supplementary Figure S1). This spike time dispersion is plotted as a function of speed on Fig. 1a right. At high speeds, the dispersions are much lower than the timescales of the input current fluctuations, which is, by definition, the signature of temporal encoding^25^.

This temporal encoding phenomenon may seem counter-intuitive and deserves an explanation. When a neuron is driven by a given fluctuating input, the output spike time dispersion depends only on the noise level, and not on the timescales of the input fluctuations^14^. In particular, without noise, the PSTH would be made of Dirac delta functions: one each time the potential reaches the threshold. Adding noise jitters the spikes, but less so if the potential’s time derivative at threshold crossing is high. This is what happens at high speed. We observe a similar qualitative change when varying contrast (Fig. 1b): temporal encoding only occurs at high contrast, and spike time dispersion decreases with contrast, as observed experimentally^26^. In addition, it can be seen on both panels that the first spike emitted after the edge stops is the most temporally precise (i.e. the first PSTH peak is sharper than subsequent peaks). Subsequent spikes suffer from jitter accumulation, again in line with experimentation^27^.

To summarize, high speeds and contrasts qualitatively change the firing behavior of each cell, increasing its temporal precision. This has consequences on synchronies when we consider a population of RGCs. For speeds from 10°/s and higher, all the ON-cells whose center area is tangential to the edge’s final position will tend to emit a first spike synchronously after the motion stops. Subsequent spikes will be progressively desynchronized.

### MSs induce synchrony – drift and tremor hardly do

Figure 1 is useful to predict what impact MSs and drift could have on RGC synchrony. Firstly, the MSs, whose speed is about 30°/s in primates^28^, should be sufficiently rapid to synchronize some of them, namely those that are strongly stimulated after the MS landing, presumably due to the presence of a contrast edge in their RFs. In addition, Fig. 1a shows that a speed of 30°/s leads to a temporal precision which is close to the theoretical maximum (corresponding to an infinite speed). Likely, the benefit of making faster MSs does not outweigh the associated metabolic costs. Secondly, the drift, whose speed is typically below 1°/s in primates^28^,^29^, is probably too slow to effectively synchronize RGCs. In the next sections, these predictions will be tested using natural images and realistic FEMs.

Finally, we investigated the effect of tremor, by animating the contrast edge in Fig. 1 with a small sinusoidal movement around its final position (data not shown). We used a frequency of 90 Hz and an amplitude of 0.5arcmin, both in the biological ranges for tremor^1^. The amplitude is thus 10-fold smaller than the radius of the RGC’s center area. Moreover, the 90 Hz frequency is strongly attenuated by the retinal low-pass filtering. Hence, the resulting oscillation in the RGC’s membrane potential had a tiny amplitude, only of about 1/10000^th^ of the difference between threshold and resting potentials. This is about 1000-fold smaller than the standard deviation of the fluctuations caused by the noise we injected in our simulations. Some experimentalists also reported slower tremor frequencies, down to 40 Hz^1^, obviously less attenuated by the retinal low pass filtering. We thus repeated the same simulation with 40 Hz instead of 90 Hz, which led to a membrane potential oscillation amplitude about 10-fold larger. However, this amplitude is still 100-fold smaller than the standard deviation of the fluctuations caused by the noise (which has the same order of magnitude as the standard deviation of the fluctuations caused by the drift+MSs with natural images, according to the simulations done in the next section). We thus neglected tremor in the rest of the study. If the real amount of noise is much lower than assumed here, and if the only motion is the tremor (no drift nor MS), then it is conceivable that the tremor-induced oscillatory current gets amplified through gain control, and thus impacts the RGCs’ firing. But this scenario is speculative, and not very ecological in any case.

### MSs allow synchrony-based representations of natural images

Let us now consider a more ecological scenario in which we examine the effects of drifts and MSs on a population of RGCs stimulated with natural images. As we will see, after each MS landing a subset of RGCs fire a volley of nearly synchronous spikes that encodes the image corresponding to the landing location.

To generate a realistic gaze trajectory, we modeled the drift as a Brownian motion, as suggested by Rucci and colleagues^30^,^31^, with a diffusion constant of 40 arcmin^2^/s^30^. In addition, MS were generated at irregular intervals using the model proposed by ref. 32 (see Methods for details). The model generates about 2 MSs per second, which consist in ballistic jumps with a mean amplitude of 30 arcmin, and a duration of 25 ms (in the biological ranges^28^). On a long timescale (>1s) these MSs avoid persistent motions, i.e. they keep the gaze close to an intended fixation position. We used these trajectories to animate natural images, and stimulated Virtual Retina with the resulting frame sequence, at 200 frames/s. We used two layers (ON and OFF) of 80 × 80 RGCs, uniformly spaced on a 4° × 4° field of view, which roughly corresponds to the primate fovea. Supplementary Video S1 illustrates the set up.

**Figure 2:**
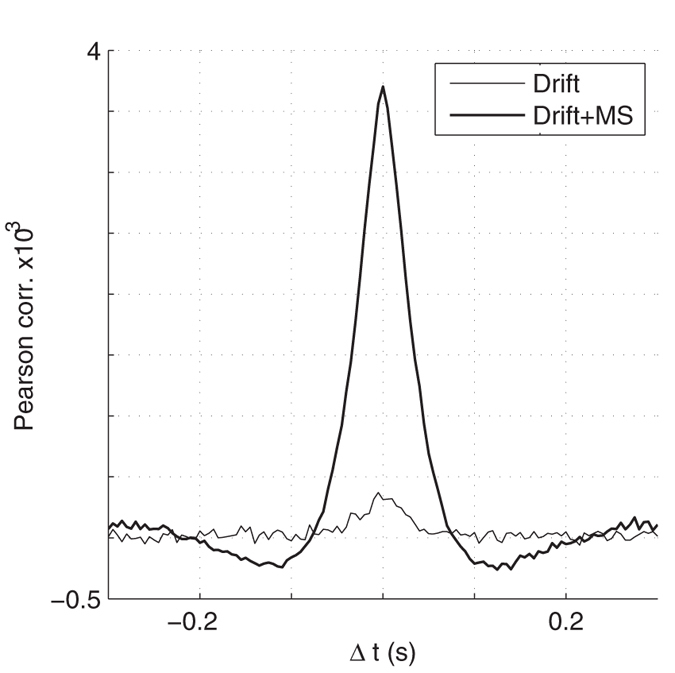
Spike crosscorrelograms (Pearson correlation coefficient, computed with a 5 ms time bin). MSs cause significant correlations at a short timescale (tens of ms and below), while the drift causes much weaker correlations.

**Figure 3:**
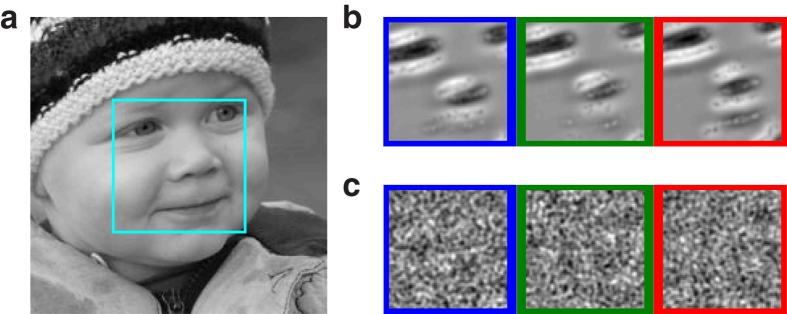
Synchrony-based representations. (**a**) Input stimulus with the 4° × 4° field of view sampled by Virtual Retina (foveal region). (**b**) Reconstructions of the downstream neurons’ preferred stimuli, after STDP-based learning. This is done by convolving the weight matrix with the RGC spatial filter (i.e. difference of Gaussians). For each downstream neuron, STDP assigned high weights to about 600 RGCs (“selected”), and zero weights the remaining ones. Notice that each reconstruction corresponds to a shifted version of the face’s salient parts: the green (respectively blue and red) neuron represents a centered (respectively shifted downward and upward) face. (**c**) Without MSs the STDP-based learning fails because the RGCs are not sufficiently synchronized.

We first computed the mean cross-correlogram between all pairs of RGCs (Fig. 2). It can be seen that the drift alone hardly synchronizes the cells (small peak), while MSs do so much more reliably (ten times higher peak). This is consistent with McCamy and colleagues’ observation^33^: they noticed that the drift moves receptive fields slowly over a small region of space, while MSs move receptive fields faster and over larger regions. They deduced that MSs are more likely than drifts to bring very dissimilar visual stimuli into the receptive fields, and thus to generate stronger stimulation, and stronger synchronizing forces. Here we find that MSs, but not drifts, are sufficient to effectively synchronize RGCs. But this raises the following questions: in the presence of MS, which RGCs fire synchronously, and when? To answer these questions, we fed the RGC spike trains to a bio-inspired unsupervised learning algorithm^34^. The algorithm uses downstream coincidence detector neurons, equipped with spike timing-dependent plasticity (STDP), and lateral inhibitory connections (please note that we do not claim that such neurons exist right after the primate retina, here it is just a way to investigate what large-scale retinal synchronous spike volleys could represent – we will come back to this point in the Discussion). For each downstream neuron, STDP progressively concentrates high synaptic weights on a small subset of RGCs that consistently fire together and discards the other ones. Lateral inhibition encourages the neurons to select different subsets. When this algorithm is trained with a single natural image, the final subsets correspond to the image’s salient parts, with different spatial shifts (see Fig. 3a,b for an example with three downstream neurons, and a face image). It is worth mentioning that, even if MSs have small amplitudes (30 arcmin on average), they can synchronize distant RGCs, because all the RGCs receive the signal change at the same time. The same algorithm failed to learn useful representations with the drift alone (Fig. 3c), presumably because synchrony was too weak in that case (Fig. 2).

**Figure 4:**
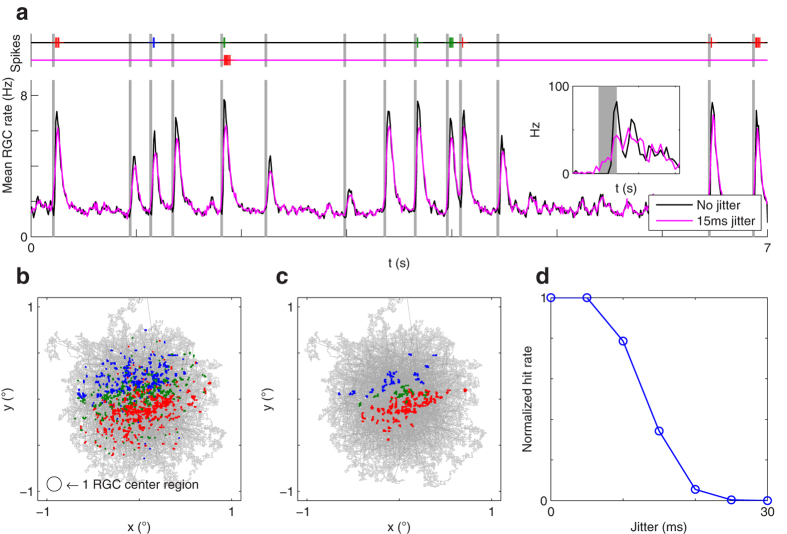
Synchronous spike patterns follow MSs and depend on the landing location. The stimulus is still the face of Fig. 3a. (**a**) Mean RGC firing rate as a function of time. Vertical gray bars indicate MSs. These are followed by strong transient activity. Red, green, and blue bars above indicate the times at which the 3 downstream neurons fire, with colors matching the ones in Fig. 3. Most of these spikes disappear when adding a 15 ms jitter. The inset shows the rate of a subset of ~150 ON-RGCs that receive a similar activation level after one MS (leading to 3 spikes in the 100 ms following the MS). (**b**) The gray line shows the gaze trajectory, made of drifts (random walk) and MSs (ballistic jumps). Colored dots indicate the positions where the 3 downstream neurons fired. These neurons fire after a MS, and each downstream neuron has a preferred landing zone. (**c**) A 15 ms jitter suppresses most of the downstream neurons’ responses. (**d**) Number of postsynaptic spikes, relative to the case with no jitter, as a function of jitter.

When do the RGC subsets fire synchronously? As a proxy for such events, one can examine the spikes emitted by the downstream neurons after learning, still using the same stimulus. These spikes occur shortly after the MSs (Fig. 4a). Different MSs activate different downstream neurons, depending on the landing location (Fig. 4b). As a control experiment, we checked that jittering the RGC spike times, by adding random delays drawn from a normal distribution (μ = 0 ms, σ = 15 ms), which preserves the spike counts but impairs synchrony, removes most of the downstream neurons’ spikes (Fig. 4a,c). With σ = 30 ms, no spike remains (Fig. 4d).

Notably, on Fig. 4a the RGC population activity following a MS does not have a series of sharp temporal peaks like in Fig. 1. This is due to an averaging effect: the population comprises cells that are differently activated by each MS, which leads to different latencies. If one restricts the analysis to RGCs that receive a similar (strong) activation level, then some sharp peaks appear (Fig. 4a inset). In other words, the strongly activated cells operate in the temporal encoding regime, which enables precise spike synchronization.

**Figure 5:**
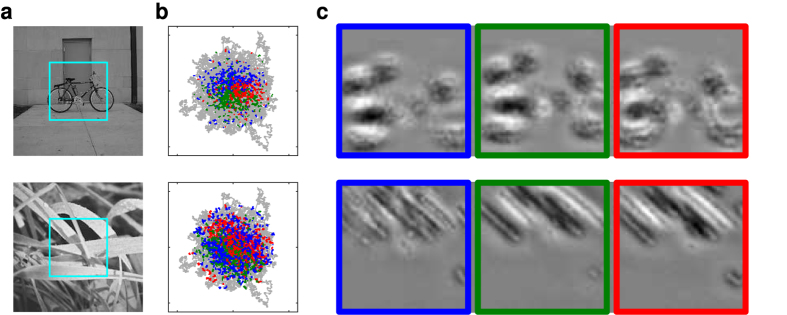
Examples of synchrony-based representations with other stimuli. (**a**) Input stimuli with the 4° × 4° field of view sampled by Virtual Retina (foveal region). (**b**) Gaze trajectory. Colored dots indicate the spikes emitted by the 3 downstream neurons, after learning. (**c**) Reconstructions of the downstream neurons’ preferred stimuli, after learning. The bike image is from the Savarese and Fei-Fei dataset^61^.

To summarize, after each MS landing a RGC subset fires synchronously with a precision of *circa* 10 ms. For a given image, the exact subset depends on the MS landing location (this implies that it also depends on the image, as we will check in the next section). Similar results can be observed with other natural images (Fig. 5). Note that the number of downstream neurons we used is arbitrary. Using more of them would lead to partitioning the landing location set more finely.

### MSs allow rapid, coincidence-based, template matching

Template matching is an operation which consists of computing the similarity between a given image patch and a stored prototype. We will now demonstrate that such a similarity can be computed from the MS-induced synchronous spike volleys, because these volleys are signatures of the corresponding image contents. Here, for simplicity, we used holistic template matching.

**Figure 6:**
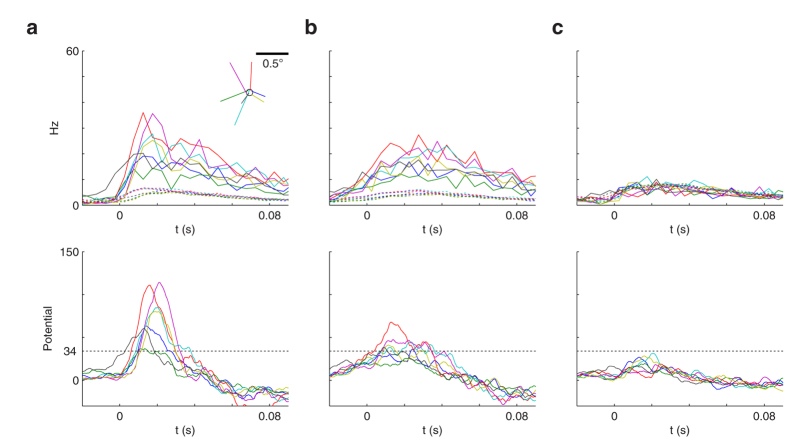
Rapid synchrony-based template matching. Here we focus on the second (green) downstream neuron of Fig. 3, which prefers MSs landing in the central zone of the face image (Fig. 4b). For illustration purposes, we used again the face stimulus, and examined the responses for 7 randomly selected MSs, landing near the center (see Methods for details) (**a**) The top plot shows the PSTH for the selected RGCs (solid lines), and for the remaining ones (dotted lines), for each MS. t = 0 is the MS landing time. Notice that the selected RGCs tend to start firing much earlier than the others, and somewhat synchronously (they also fire more spikes). The inset shows the MS trajectories, which land in a neighborhood of the central position (circle). The bottom plot shows the downstream neuron’s unconstrained potential (ignoring the threshold). Most MSs generate a high potential peak due to nearly coincident spikes from selected RGCs. (**b**) With a 15 ms jitter, the selected RGCs’ response onsets are more spread out, which flattens the potential peaks. (**c**) As in (**a**) but using a different stimulus (the bike image in Fig. 5a). The selected RGCs no longer fire synchronously. Resulting potential peaks are weak. The horizontal doted line on bottom plots is a hypothetical threshold of 34, which would lead to no false alarm with the bike image, and no miss with the face image, despite strong MS-to-MS variability.

We found that the potential of each downstream neuron shortly after the MS landing may be interpreted as the similarity between the stimulus seen from the landing location and the neuron’s preferred stimulus. To give an example, we examined the responses of the green neuron shown in Figs 3 and 4. This neuron prefers MSs that land near the central position of the face image (Fig. 4b). For illustration purposes, we selected seven of such MSs, and plotted the mean PSTH for all the selected RGCs and for the remaining ones (Fig. 6a top, solid and dotted lines respectively). The plot shows that the selected RGCs tend to emit their first spikes synchronously. The time at which they do so is variable from one MS to another (~10 ms after the MS landing on average). These volleys of synchronous spikes, arriving through strong synapses, cause high peaks in the downstream neuron’s potential (Fig. 6a bottom), with a latency of only ~20 ms. The remaining RGCs tend to fire fewer spikes, do so much later and more asynchronously. Unsurprisingly, adding a 15 ms jitter spread out the first spikes, which flattened the potential peaks (Fig. 6b).

Is the subset of synchronous RGCs stimulus-specific? To answer this question, we examined the responses of the green neuron, trained with the face image, to the bike image shown in Fig. 5a. In that case, the responses of the ~600 selected RGCs are statistically indiscernible from that of the remaining ones (Fig. 6c): late, weak, and asynchronous (actually, another small RGC subset corresponding to the bike’s salient parts is synchronized, but it is hidden in the huge population of 12,800 RGCs). The resulting potential peaks are weak.

To summarize, the downstream coincidence detector neuron robustly discriminates between the two stimuli in just ~20 ms, despite strong MS-to-MS variability in the RGC responses that is due to different retinal images before each MS. It is worth mentioning that the response magnitude only depends on how many of the selected RGCs spike synchronously, and on the precision of this synchrony. The downstream neuron ignores the MS landing time, or if the spike volley it is integrating corresponds to the first spikes. In practice, however, the most synchronous volley is the first spike volley (as shown in Fig. 1).

## Discussion

Referring to Marr’s three levels of analysis^35^, we may say that the nature of the *computation* we have considered here is that of template matching between an input image patch and a stored prototype. A possible *algorithm* to do so, then consists in convolving the image with a battery of filters, selecting the most active units, and comparing this subset with the one corresponding to the prototype. As far as the *implementation* level is concerned, the filtering is handled by the RGCs. But how is it then that downstream neurons are able to identify the most active ones? There are three possibilities: it could be by exploiting the fact that these RGCs fire more spikes (rate coding); or in the presence of MSs, that these RGCs fire earlier (relative latency, rank-order coding); or finally, that these RGCs fire more synchronously (synchrony-based coding). We argue that this last possibility has not attracted enough attention, yet it has four main advantages. (I) The readout is rapid: a decision can be made as soon as the first spikes are emitted, about 20 ms after the MS landing. (II) It only needs coincidence detector neurons, whereas decoding ranks requires other mechanisms such as shunting inhibition^36^ or winner-take-all^37^, for instance. (III) There is no need for a reference time: the knowledge of the MS landing time is not required. And (IV), the required connectivity can spontaneously emerge with STDP.

Note that we did not try to quantify the amount of synchrony provided by the MSs. This amount strongly depends on the MS rate, which itself depends on attention, proximity of the last saccade etc., as well as on other MS and retinal parameters, whose values are debated. Instead, we deliberately remained qualitative: from Fig. 2, it is clear that MS induce precise (10–20 ms) synchrony, whereas the drift hardly does so.

Importantly, we do not claim that the readout used here is realistic. We used a holistic 4° × 4° template matching task to demonstrate the capacity of synchrony-based coding in the retina. Yet in the primate visual system such template matching is not done in one step from the RGCs’ spike trains. Instead, the input is processed through the ventral stream where information from distant regions is progressively integrated. This raises important questions: can the MS-induced synchrony propagate across the different areas, and if so, does it really play a role in the computations? Retinal synchrony propagates at least until the LGN^38^,^39^. Indeed, conduction velocities from the RGC to the LGN cells, which depend on axon diameters, are faster for signals coming from more peripheral portions of the retina^40^, which exactly compensates the additional distance to be travelled, and thus provides for equal time-of-arrival at the LGN irrespective of the retinal site that is stimulated. This strongly suggests that RGC synchrony matters, at least for LGN cells. In the primary visual cortex (V1) cells presumably gain their orientation selectivity through thalamic synchrony^39^. In particular, MS-induced synchrony may enhance spatial summation in V1^1^,^41^. Under specific conditions synchrony could in principle propagate to higher order visual areas^12^,^42^,^43^, and the prevalence of strong feedforward inhibitory circuits throughout the central nervous system suggests that synchrony codes may be widespread^13^. Yet direct evidence for it beyond V1 has been lacking. One difficulty to detect synchrony coding is that all the involved cells must be recorded at the same time (high trial-to-trial variability in the spike times with respect to the stimulus onset does not rule out synchrony coding^14^,^44^). Finally, it is also conceivable that synchrony coding is used up to V1, and then converted into rate coding for further processing.

Of course retinal responses are location-dependent (i.e. not shift-invariant). This is why the downstream neurons learned shifted versions of a same stimulus (Figs 3 and 5). The issue of how shift-invariance is progressively gained along the ventral stream is out of the scope of the current paper, which focuses on the retinal code (nevertheless it has been hypothesized that a maximum operation could help^45^, and this can be implemented easily with temporal codes^46^). We did not address either the question of how receptive fields emerge. The learning procedure that we introduced should not be interpreted as a way to mimic how real neurons become selective to specific features. We trained the algorithm on one image at a time to extract the MS-induced synchronies obtained with that particular image. Again it is just a way to investigate what large-scale synchronous retinal spike volleys represent.

Does synchrony really impact perception, or is it an epiphenomenon? One solution to address this question is to disrupt neural synchronies, and examine the consequences on perception. This can be done by splitting a stimulus into multiple parts, and presenting these parts asynchronously. Using this approach, Greene has shown that delays in the millisecond range between successively presented dots marking the boundary of a shape can impair recognition in humans^47^. In addition, it has been shown that when stimulating a rat’s visual cortex with two asynchronous pulses, arriving through two different electrodes, the animal can detect and report timing differences of only 15 ms^48^. Synchrony, or lack thereof, thus has perceptual consequences.

According to our first set of simulations (Fig. 1), rapidly moving stimuli create synchrony among RGCs, even without eye movements. However, static or slowly moving ones do not, and this is when MSs come in handy. MSs are able to reconcile static and slowly moving stimuli with short neuronal integration and STDP timescales, thereby enabling one-step feedforward readout, much like brain oscillations^49^, and thus could enhance the perception of such stimuli.

In this paper, we focused on MSs. Yet other kinds of motions with high speed and abrupt stops, for example saccades, nystagmus, or head motions, may also synchronize RGCs. These motions, however, are typically larger and thus also serve another purpose: to bring a selected target to the fovea. Conversely, the main function of MSs could be to provide, when needed, discrete snapshots of our foveal field of view’s high spatial frequency content, using a synchrony-based code. In addition, even single RGC spatial resolution is presumably improved during MSs^50^. Both phenomena probably explain why we do more MSs when paying attention to fine spatial details^7^,^8^. We also speculate that less frequent and/or slower MSs in healthy aging or in certain pathologies such as amblyopia could explain some perceptual deficits, in particular with high spatial frequencies, and thus could cause lower visual acuity and vernier acuity.

Of course, further visual computations certainly occur between MSs, based on the RGCs’ sustained asynchronous responses, as well as on recurrent processing. Indeed, from Fig. 4, it can be estimated that asynchronous spikes represent about 2/3 of the total number of spikes, and thus these asynchronous spikes certainly have important functions, which we did not investigate in this study. The drift certainly plays a key role here: it has been shown that it removes the predictable image correlations, and thus could enhance the departures from these predictions^30^.

One limitation of the Virtual Retina software that we used in this study is that it does not involve any microsaccadic suppression mechanism. In real retinas, it is known that amacrine cells (not included in Virtual Retina), which are excited by the global motion signal during saccades, inhibit the RGCs^51^,^52^. It is conceivable that this also occurs during MSs. This would remove some variability by ensuring all the RGCs are in the same state when the MS stops, whatever their own history. As a result, synchrony would be even more precise. Amacrine cells may also cause the synchronized oscillatory activity^53^ which has been observed experimentally^54^. Including them in our simulations should enhance and extend the MS-induced synchronizations, which would also recruit more RGCs. This should in fact improve synchrony-based coding, providing the recruited cells are still minority. This will be investigated in future work.

As stated above, the retinal simulator (Virtual Retina) used in this study has been validated using various experimental datasets. We are thus confident that the MS-induced synchrony we showed here is not an artifact of the model but also occurs in real retinas, at least from a qualitative point of view. Nevertheless, the results presented here should be confirmed experimentally and this could prove to be challenging. Firstly, to observe the qualitative change in the firing behavior of a RGC (Fig. 1), one needs time bins in the millisecond range, and therefore hundreds, or even thousands of trials per speed and contrast condition, resulting in very long retinal recordings. Under those conditions, some non-stationary factors (e.g., bleaching of the photoreceptors in *ex-vivo* experiments, or changes in the physiological state of the living tissue) could slightly affect the RGC responses, for example by delaying the latencies with a few milliseconds and/or increasing their dispersion. This would smooth the PSTH peaks and might hide the qualitative change. Secondly, to study the impact of FEM on synchrony in a population of RGCs using the same methodology as here, one would need at least hundreds of MSs per landing zone for each stimulus, and so thousands of them in total, which means retinal recordings lasting thousands of seconds. Again, possible non-stationary factors could be an issue. Natural images pose an additional challenge. Current techniques do not allow one to exhaustively record all the RGCs in a given retinal patch, therefore some RGCs whose RFs fall on edges might be unrecorded, and we would not detect their synchronous spike volleys. An intermediate step could be to use an artificial stimulus with a repeating motif, and to gain power by averaging the responses across many cells (as opposed to many MSs), using a dense Multi-Electrode Array (MEA) such as the Active Pixel Sensor CMOS MEA consisting of 4096 electrodes spanning an active area of 2.67 × 2.67 mm^55^,^56^.

## Methods

We used the open source Virtual Retina simulator available here:

http://www-sop.inria.fr/neuromathcomp/public/software/virtualretina/

Our Matlab code for eye movements, and STDP-based learning has been made available on ModelDB:

http://senselab.med.yale.edu/modeldb/showmodel.cshtml?model=188423.

### Virtual Retina

The simulator is highly configurable through a xml parameter file, and we used parameters corresponding to primate foveal midget cells (according to ref. 15), gathered in Table 1. The reader is referred to ref. 15 for further information about the model.

### Fixational eye movements (drift & microsaccades)

The drift was modeled as a Brownian motion, as proposed by ref. 30. In practice, the Brownian motion is approximated by a random walk. At each time step, the gaze moves in one of the four possible directions (up, down, left, right), picked randomly, by an amount:


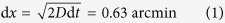


where d*t* = 5 ms is the time step, and D is the diffusion constant of the Brownian motion, estimated at 40 arcmin^2^/s in humans^30^. The motion thus occurs on a 2D lattice with a spatial step d*x*.

As far as the MSs are concerned, we used a variation of the FEM model proposed by ref. 32. Briefly, the model is inspired by a phenomenology of a walk in a swamp, where the ground under a walker located at lattice site (i, j) sinks at each time step:


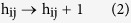


while all non-occupied sites relax to the steady zero level:


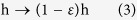


In addition, if the local h is greater that some threhold h_c_ then a MS (ballistic jump) is generated, towards the site which minimizes h + u + u_1_, where u is a quadratic potential, which encourages the walker to remain close to the central position, and u_1_ encourages vertical and horizontal MSs, as opposed to oblique (Note that here, in contrast with Engbert and colleagues’ model, neither h nor u influenced the drift direction, which, as stated above, was picked at random. This leads to a Brownian motion for the drift, which seems realistic, and allows to use a diffusion constant measured in humans^30^).

This model reproduces a number of MS statistics^32^. However, at least with the parameters suggested in the paper, we found that it generated too small saccades (less than 10d*x* = 6 arcmin), thus we had to adjust the model.

The main modification we made concerns the sinking (operation 1 above). Now, not only the ground located below the walker sinks, but also the ground located in a neighborhood of the walker. More specifically, the sinking is proportional to 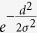, where *d* is the distance to the walker, and *σ* = 2. This resulted in larger MSs, with a mean amplitude of ~30 arcmin, which is in the biological range^28^.

The other parameters are given in Table 2.

### STDP & coincidence detector neurons

We used the competitive STDP-based learning algorithm of ref. 34, with the parameters given in Table 3. The algorithm uses classic additive STDP, with exponential windows, and the nearest spike approximation. We used τ^−^ = τ^+^ and a^−^ = −a^+^ so as not to favor/disfavor inputs which fire more.

We added a homeostatic term^57^: w^out^  <0 is added to all synaptic weights at each postsynaptic spike. This penalizes neurons that fire too much. More negative values for w^out^ result in fewer selected synapses after learning.

Neurons are modeled with Gerstner’s spike response model (SRM)^58^. The kernel we used for the excitatory postsynaptic potential (EPSP) is based on the notion of effective signal, in the presence of a fast adapting threshold (see ref. 59). It is defined as the difference between the fast adapting threshold, with time constant τ_thr_, and the membrane potential:


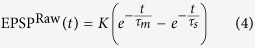



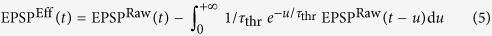


Constant K was chosen such that:


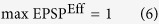


The neurons detect spike coincidences at a timescale defined by τ_thr_, which is presumably around 5 ms in cortex^59^. We found it useful to use STDP time constants that roughly match this timescale (τ^+^ = τ^−^ = 3 ms). We admit, however, that these timescales are faster than what most experimentalists report, at least *in vitro*.

For each natural image, we trained the neurons for 10^5^ seconds (biological time), to make sure all the synaptic weights were close to 0 or 1.

Concerning the computation with the drift only (Fig. 3c), we had to lower T to get postsynaptic spikes. On Fig. 3c, T = 40. An exhaustive parameter search for T gave similar results (i.e., the neurons stopped firing after a while, indicating that no repeating spike pattern was found).

### PSTHs

The PSTHs in Fig. 1 were computed using 10^5^ trials, and a time bin of 4 ms, and fitted with Gaussian mixture models (see Supplementary Figure S1). A time bin of 10 ms was used in Fig. 4a (4 ms for the inset), and of 5 ms for Fig. 6.

### Cross-correlograms

The stimulus was the face image in Fig. 3a. To save computation time, we randomly selected 5000 RGC pairs only, among the set of possible pairs (whose cardinal is N(N-1)/2~10^8^, where N = 80 × 80 × 2 is the number of RGCs). For the drift only case, we took the spike trains obtained with drift + MSs, extracted the drift periods (from last MS onset + 300 ms to next MS onset), and concatenated these periods (another option would have been to use a pure random walk, but the problem is then that the gaze can drift forever, beyond the image boundaries).

### Template matching

For Fig. 6, we randomly selected 7 MSs landing at less than 0.05° of the center (see circle on panel a’s inset). Unconstrained potentials were obtained by convolving the spike trains from selected RGCs with the above-mentioned effective EPSP.

## Additional Information

**How to cite this article**: Masquelier, T. *et al*. Microsaccades enable efficient synchrony-based coding in the retina: a simulation study. *Sci. Rep*. **6**, 24086; doi: 10.1038/srep24086 (2016).

## Supplementary Material

Supplementary Information

Supplementary Video S1

## Figures and Tables

**Table 1:** Parameters for Virtual Retina simulations representing midget cells in the foveal region of a primate retina (see ref. 15).

Parameter	Value	Comment
**Outer Plexiform Layer**		
σ_C_	0.05°	Centre gaussian’s sigma
τ_C_	10 ms	Centre signal low pass filtering time constant.
τ_U_	100 ms	Undershoot high pass filtering time constant.
w_U_	0.8	Undershoot transient relative weight.
σ_S_	0.15°	Surround gaussian’s sigma
τ_S_	4 ms	Surround signal low pass filtering time constant.
λ_OPL_	10 Hz/Lum. unit	Overall gain of the centre-surround filter.
w_OPL_	1	Relative weight of centre and surround signal.
Use leaky heat equation	True	Averaging by gap junctions rather than dendritic spread. Leads to a non-separable spatio-temporal filter, but somewhat more realistic.
**Bipolar Cells**		
λ_OPL’_	50	Another gain applied right after *λ*_*OPL*_, thus without biological meaning, but useful for implementation issues.
g_A_^0^	50 Hz	Inert leaks in membrane integration.
σ_A_	0.2°	Size of the spatial neighbourhood used to estimate local contrast.
τ_A_	5 ms	Size of the temporal neighbourhood used to estimate local contrast.
λ_A_	0 Hz	Strength of the gain control feedback loop (no contrast gain control in primate midget cells)
**Inner Plexiform Layer**		
τ_G_	20 ms	High pass filtering time constant.
w_G_	0.7	Transient relative weight.
σ_G_	0°	No additional pooling for midget cells.
v_G_^0^	0	Bipolar linear threshold.
λ_G_	100 Hz	Slope in the linear area.
i_G_^0^	37 Hz	This is below the threshold current (50 Hz). Thus in the dark the threshold is reached only because of the noise (see below), which leads to a irregular Poisson-like spontaneous activity (at ~1 Hz).
**Retinal Ganglion Cells (RGC)**		
g_L_	50 Hz	Leak conductance (thus the membrane time constant is 20 ms)
σ_v_	0.1	Gaussian white noise current’s normalized amplitude. Integration of this current by the RGCs leads to a Gaussian auto-correlated process with time constant 1/g^L^ and variance σ_v_.
η_refr_	3 ms	Refractory period
Density	20 cells/°	RGC density (for each polarity). That is a mean inter-RGC interval of 0.05°

**Table 2:** Parameters for fixational eye movement trajectory generation using the model by Engbert *et al*. (see ref. 32).

Parameter	Value	Comment
L	401	Grid size
ε	2.5e-05	Relaxing factor
h_c_	87	Threshold for MS generation
λ	1	Potential slope parameter
χ	0.12	Oculomotor potential slope parameter

**Table 3:** Parameters for STDP & SRM neurons (see ref. 34).

Parameter	Value	Comment
τ^+^	3 ms	LTP time constant
τ^−^	3 ms	LTD time constant
a^+^	2^−8^	LTP learning rate
a^−^	−a^+^	LTD learning rate
w^out^	−0.0015a^+^	Homeostatic term
τ_thr_	3 ms	Adapting threshold time constant
τ_m_	20 ms	Membrane time constant
τ_s_	2 ms	Excitatory synapse time constant
τ_i_	5 ms	Inhibitory synapse time constant
α	2.0	Inhibition strength
T	60	Threshold
