# Supplementary material for: Microsaccades enable efficient synchrony-based coding in the retina: a simulation study
Source: Sci Rep. 2016 Apr 11;6:24086. doi: 10.1038/srep24086 (PMC4827057; doi:10.1038/srep24086)
Supplement: Supplementary Information [file srep24086-s1.pdf]

Microsaccades enable efficient synchrony-based coding in the retina: a simulation study.

Timothée Masquelier, Geoffrey Portelli, Pierre Kornprobst

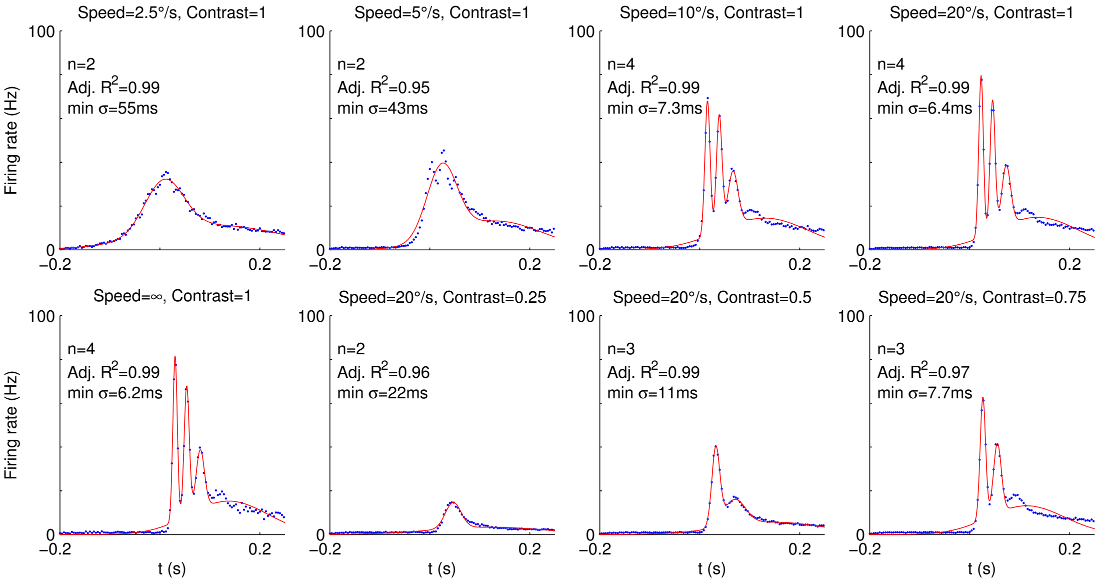

Supplementary Figure S1. Estimating the spike time dispersions by fitting the PSTHs with Gaussian mixture models. For each condition, the number of Gaussians we used *n* is the minimal number for which the goodness-of-fit parameter "Adjusted R^2" is greater than 0.95. Then we defined the temporal dispersion as the minimum of the Gaussians' sigmas (which in practice corresponds to the earliest peak).

Supplementary Video S1. Stimulus and resulting RGC activity. Panel "Trajectory" plots the gaze direction in degrees. Panel "Input video" represents the retinal image. Panel "RGC spikes" shows the spikes emitted by ON- and OFF-center RGCs, respectively in white and black. Notice that after each MS there is a volley of synchronous spikes corresponding to the stimulus edges. Panel "Mean RGC rate" shows the population activity. The red line indicates the current time.
